# The Use of Antifibrotic Recombinant nAG Protein in a Rat Liver Fibrosis Model

**DOI:** 10.1155/2019/9846919

**Published:** 2019-06-02

**Authors:** Maha M. Arafah, Mohammad M. Al-Qattan, Durria A. Abdulmaged-Ahmed, Ghada A. Al-Nafesah, Nessrin Y. Jadu, Reginald S. Bagayawa, Medhat K. Shier, Amir Marzouk, Hend S. Almalki

**Affiliations:** ^1^Department of Pathology, King Saud University, Riyadh, Saudi Arabia; ^2^Department of Surgery, King Saud University, Riyadh, Saudi Arabia; ^3^College of Medicine Research Center, Deanship of Scientific Research, King Saud University, Riyadh, Saudi Arabia; ^4^Department of Surgery, King Faisal Specialist Hospital and Research center, Riyadh, Saudi Arabia; ^5^Department of Surgery, National Guard Health Affairs, Riyadh, Saudi Arabia; ^6^Department of Surgery, Prince Sultan Military Medical City, Riyadh, Saudi Arabia

## Abstract

**Objectives:**

The “nAG” protein is the key protein mediating the regeneration of amputated limbs in salamanders. The senior author (MMA) developed the original hypothesis that since “nAG” is a “regenerative” protein, it must be also an “antifibrotic' protein. The antifibrotic properties were later confirmed in a rabbit skin hypertrophic scar model as well as in a rat spinal cord injury model. The aim of this study is to evaluate the potential therapeutic properties of the nAG protein in a rat liver fibrosis model.

**Methodology:**

Liver fibrosis was induced using intraperitoneal injections of carbon tetrachloride (CCL4). A total of 45 rats were divided equally into 3 groups: Group I (the control group) received normal saline injections for 8 weeks, Group II received CCL4 for 8 weeks, and Group III received CCL4 and nAG for 8 weeks. At the end of the experiment, the serum levels of 6 proteins (hyaluronic acid, PDGF-AB, TIMP-1, laminin, procollagen III N-terminal peptide, and collagen IV-alpha 1 chain) were measured. Liver biopsies were also taken and the stages of live fibrosis were assessed histologically.

**Results:**

The CCL4 treatment resulted in a significant increase in the serum levels of all 6 measured proteins. The nAG treatment significantly reduced these high levels. The degree of liver fibrosis was also significantly reduced in the CCL4/nAG group compared to the CCL4 group.

**Conclusions:**

nAG treatment was able to significantly reduce the serum levels of several protein markers of liver fibrosis and also significantly reduced the histological degree of liver fibrosis.

## 1. Introduction

Salamanders (which are lower vertebrates) are known to regenerate their amputated limbs. The “nAG” protein (nAG stands for newt Anterior Gradient) is the key protein mediating this form of regeneration [1]. The amputation stump of the salamander forms a blastema (a mound of proliferating mesenchymal cells) in which nAg is expressed. The nAG protein is expressed by Schwann cells of regenerating axons and peaks at 5–7 days postamputation. At 10–12 days, the protein is also expressed in glands in the dermis underlying the wound epithelium [1].

The senior author (MMA) developed the original hypothesis that since “nAG” is a “regenerative” protein, it must also be an “antifibrotic” protein. Hence, a new nAG gene (suitable for higher vertebrates including humans) was designed, synthesized, and cloned. The cloned vector was successfully transfected into human fibroblasts. nAG expression was found to suppress the expression of collagen in human fibroblasts regardless of the presence of Transforming Growth Factor Beta (TGF*β*) [2]. The antifibrotic properties of the nAG protein were later shown in several animal models such as a rabbit model of hypertrophic scar [3], a mouse model of digital tip amputation [4], and a rat spinal cord crush injury model [5]. The antifibrotic effects of nAG on liver fibrosis have not been previously investigated.

Liver fibrosis may be induced by several factors such as nonalcoholic fatty infiltration of the liver, alcoholic liver disease, viral hepatitis, autoimmune hepatitis, toxin-induced hepatitis, and hereditary metabolic diseases [6, 7]. The end result of liver fibrosis is cirrhosis leading to portal hypertension, liver failure, and the increased risk of hepatocellular carcinoma. The only effective treatment of decompensated liver cirrhosis is liver transplantation [6, 7]. Fibrogenesis and organ fibrosis are mediated by different cells in different organs such as the fibroblast in the skin [3], the astrocyte in the spinal cord [5], and the stellate cell in the liver [8].

In the current study, we investigated the potential therapeutic properties of the nAG protein in liver fibrosis using a rat model.

## 2. Methodology

This study was approved by our institutional review board and was conducted according to the Guidelines for Animal Experiments (Project# E-13-926).

### 2.1. The Animal Model

We induced liver fibrosis in rats using carbon tetrachloride (CCL4) and this is a well-known model of rodent liver fibrosis [9, 10]. A total of 45 adult Sprague-Dawley rats (weighing between 240-260 gm) were divided into three groups (15 rats in each group): Group I (Control group) received twice weekly intraperitoneal injections of normal saline for 8 weeks, Group II received twice weekly intraperitoneal injections of CCL4 (0.5 ml/kg of the 25% CCL4 solution) for 8 weeks, and Group III received twice weekly intraperitoneal injections of CCL4 (0.5 ml/kg of the 25% CCL4 solution) as well as 2 ug of the recombinant nAG protein for 8 weeks.

### 2.2. ELISA Assessment of Protein Markers

At the end of the experiment, the serum level of six protein markers of liver fibrosis were measured in all rats using ELISA: hyaluronic acid, Platelet Derived Growth Factor-AB (PDGF-AB), Tissue Inhibitor of Metalloproteinase-1 (TIMP-1), laminin, procollagen III N-terminal peptide (PIII-NP), and collagen type IV-Alpha 1 chain (collagen 4-*α*1).

### 2.3. Histopathological Assessment

Furthermore, liver biopsies were taken from all rats at the end of the experiment (8 weeks) and the histological grading of liver fibrosis was graded using the Metavir scoring system [11] as shown in Table 1. All biopsies were stained with both Hematoxylin and Eosin (H&E) and Elastic fiber-Verhoeff's Van Gieson (EVG) stains. The grading was done by a Consultant Histopathologist who was blinded to the rat groups.

### 2.4. Statistical Analysis

Statistical analysis was performed using Statistical Package for the Social Sciences (SPSS) version 22.0 software (SPSS Inc., Chicago, Il, USA). For the study of serum protein levels, the means and Standard Deviations (SD) were calculated for the three groups (the control and the two experimental groups). We used the one-way analysis of variance (ANOVA) to compare the three groups and the post hoc test Dunnett T3 for multiple comparisons between the groups. P-value of < 0.05 was considered significant. For the histological grading of liver fibrosis, we compared the percentages of no/mild fibrosis versus moderate/severe fibrosis in the two experimental groups using the Fisher's exact/Chi-square tests. P-value of < 0.05 was considered significant.

## 3. Results

### 3.1. ELISA Assessment of Protein Markers

The results of the serum levels of 6 proteins in three groups are shown in Table 2.

#### 3.1.1. Hyaluronic Acid

Hyaluronic acid doubled in the CCL4 group compared to the control group (P<0.05 between groups I & II) and the nAG treatment was able to normalize its serum levels with no significant difference between the control and the CCL4/nAG group.

#### 3.1.2. PDGF-AB

The serum levels of PDGF-AB increased more than 10-fold in the CCL4 group compared to the control group (P<0.05 between groups I & II). The nAG treatment was able to significantly reduce (but not normalize) the serum levels of PDGF-AB. Hence, there was a significant difference (P<0.05) between groups I & III and also between groups II & III.

#### 3.1.3. TIMP-1

The serum levels of TIMP-1 increased more than 12-fold in the CCL4 group compared to the control group (P<0.05 between groups I & II). The nAG treatment was able to significantly reduce (but not normalize) the serum levels of TIMP-1. Hence, there was a significant difference (P< 0.05) between groups I & III and also between groups II & III.

#### 3.1.4. Laminin

The serum levels of laminin increased almost 5 folds in the CCL4 compared to the control group (P<0.05 between groups I & II). The nAG treatment was able to significantly reduce (but not normalize) the serum levels of laminin. Hence, there was a significant difference (P<0.05) between groups I & III and also between groups II &III.

#### 3.1.5. PIII-NP

The serum levels of PIII-NP were significantly increased in the CCL4 group compared to the control group (P<0.05 between groups I & II). The nAG treatment significantly reduced the serum level of PIII-NP below levels of the control group. Hence, there was a significant difference (P<0.05) between groups I & III and also between groups II & III.

#### 3.1.6. Collagen 4-*α*1

Finally, the serum levels of collagen 4-*α*1 increased more than 7 folds in the CCL4 group compared to the control group (P<0.05 between groups I & II). The nAG was able to normalize the serum levels of collagen 4-*α*1. Hence, there was no significant difference between the control and the CCL4/nAG groups.

### 3.2. Histopathological Assessment

As expected, all rats in the control group (n=15) had no liver fibrosis and hence, these rats were not included in the statistical analysis of the grading of liver fibrosis.  Table 3 summarizes the results of the staging of liver fibrosis between two experimental groups. The nAG treatment significantly reduced the histological degree of liver fibrosis (P= 0.008).

## 4. Discussion

Our study is the first investigation of the therapeutic potential of the antifibrotic nAG protein in liver fibrosis. nAG treatment was able to significantly reduce the serum levels of several protein markers of liver fibrosis [12] and also significantly reduced the histological degree of liver fibrosis. nAG was injected intraperitoneally in our experiment. From the clinical point of view, drugs targeting organ fibrosis should consider the location of the primary profibrotic cells of the organ (the stellate cells in the liver). These cells are located in the space of Disse between the endothelial cells and hepatocytes. Hence, nAG may be more effective if combined with another carrier peptide to pass through the sinusoidal endothelial barriers and also to escape the uptake by the Kupffer cells and hepatocytes [13, 14].

The pathophysiology of liver fibrosis is well described in the literature [6]. In normal livers, the stellate cells are quiescent and they function to store vitamin A (seen histologically as intracellular retinoid droplets) and to regulate sinusoidal blood flow. After the liver insult (such as pathogens, abnormal fatty infiltration, drugs, toxins, and free radicals) the inflammatory reaction results in the release of TGF*β* and several cytokines/chemokines including the interleukins IL-1B and IL-6 [6]. This results in the activation of stellate cells. Activated stellate cells lose their retinoid droplets, proliferate, transform into myofibroblasts, and release TGF*β*, resulting in further cell activation. Activated stellate cells also release PDGF (inducing further stellate cell proliferation) and extracellular matrix proteins such as collagens, hyaluronic acid, and laminin. The abnormally high level of collagen is not only due to increased collagen production, but also due to decreased collagen degradation since the production of TIMP1 is also increased [15]. The nAG treatment was able to significantly reduce the serum levels of TIMP-1 (Table 2).

Our study also showed that the nAG treatment normalized the levels of hyaluronic acid (Table 2). Hyaluronic acid is mainly synthesized in the liver and is an important component of the liver extracellular matrix. It is normally degraded in the sinusoides by the hyaluronidase enzyme. The serum level of hyaluronic acid is known to correlate with the degree of liver fibrosis in both alcoholic and nonalcoholic liver diseases [16].

The effectiveness of nAG in reducing the production of collagen production has been shown in human skin fibroblast [2]. In fibroblasts, the effect on collagen III was more pronounced than the effect on collagen I [2]. Hence, it is of no surprise that nAG treatment in the current experiment was able to significantly reduce the serum levels of PIII-NP below the normal control levels (Table 2). PIII-NP is formed during the synthesis of collagen III. The serum level of PIII-NP is a known marker for liver fibrosis [17].

The nAG treatment in our study was also able to normalize the serum levels of collagen 4-*α*1 which is also a marker for liver fibrosis [18]. Collagen type IV is a main component of the basement membrane. In liver fibrosis, there is excessive remodeling of the basement membrane and excessive release of its peptide fragments (such as collagen 4-*α*1) in the circulation [19].

Laminin in the liver is found both in the basement membrane (where it is associated with collagen type IV) as well as in the extracellular matrix (where it is associated with collagen types I & III) along the fibrous septa and within the space of Disse [20]. Hence, the serum levels of laminin correlate with the degree of liver fibrosis [20]. Our study showed that the nAG treatment is able to significantly decrease the serum levels of laminin in experimental rats (Table 2).

There are three important PDGF isoforms: PDGF-AA, PDGF-AB, and PDGF-BB. PDGF-AA selectively binds to PDGF-receptor alpha, while the latter two isoforms bind to both alpha and beta receptors of PDGF [21]. In abnormal skin fibrotic disorders, all isoforms mediate myofibroblast proliferation and excessive collagen production [22]. In the normal liver, quiescent stellate cells express only the alpha receptors of PDGF. Hence, PDGF-AA is thought to mediate the normal functions of the quiescent stellate cells. Once stellate cells get activated, they express the beta receptors of PDGF [23, 24]. Hence, several authors have studied the serum levels of PDGF-BB in patients with liver fibrosis/cirrhosis. Zhou et al. [25] found a significant negative correlation between the serum levels of PDGF-BB and the liver fibrosis stage, while a significant positive correlation was noted by Zhang et al. [26]. These contradicting results may be related to the fact that the extrahepatic concentration of PDGF-BB is known to be related to platelet count. Hence, cirrhotic patients with thrombocytopenia will have a tendency for lower levels of serum PDGF-BB [27, 28]. To our knowledge the serum levels of PDGF-AB have never been previously studied in liver fibrosis/cirrhosis in the clinical or in the experimental setting. Our study showed that the CCL4-induced liver fibrosis model resulted in more than 10-fold increase in the serum levels of PDGF-AB and that nAG treatment significantly reduced these elevated levels (Table 2).

Many drugs have been proposed for the management of liver fibrosis [29–33]. We believe that nAG will eventually be included in the list of management protocols of fibrotic liver disease.

## 5. Conclusions

nAG treatment was able to significantly reduce the serum levels of several protein markers of liver fibrosis and also significantly reduced the histological degree of liver fibrosis. Further studies are required to investigate the effect of nAG if combined with a carrier peptide to selectively increase its concentration in the stellate cells responsible for the fibrotic reaction in the liver.

## Figures and Tables

**Table 1:** Metavir histological grading of liver fibrosis.

Grade of fibrosis	Description
F0	no fibrosis

F1	Mild fibrosis: Fibrous portal expansion with mild localized fibrosis in the portal area

F2	Moderate fibrosis: Portal fibrosis with few fibrous septa.

F3	Severe fibrosis: Portal fibrosis with numerous fibrous septa.

F4	Cirrhosis: Marked portal-to-portal and portal-to central fibrosis with regenerative nodules.

**Table 2:** Results of the serum levels of various proteins in the three groups.

SERUM LEVEL OF:	Group I	Group II	Group III
	(Control)	(CCL4 treatment)	(CCL4/nAG treatment)
	N=15	N=15	N=15
Hyaluronic acid	15.133 ± 0.063 *∗*	29.80 ± 6.145 *∗*^∧^	15.739 ± 3.231 ^∧^

PDGF-AB	18.292 ± 0.023 *∗*+	202.839 ± 124.73 *∗*^∧^	58.905 ± 29.198 ^∧^+

TIMP-1	35.563 ± 0.0299 *∗*+	449.25 ± 294.71 *∗*^∧^	161.919 ± 67.518 ^∧^+

Laminin	257.27 ± 12.91 *∗*+	1238.588 ± 622.419 *∗*^∧^	415.34 ± 136.934 ^∧^+

Procollagen III N-terminal peptide	0.242 ± 0.021 *∗*+	0.355 ± 0.050 *∗*^∧^	0.161 ± 0.051^∧^+

Collagen Type IV- Alpha-1 chain	2.933 ± 2.193 *∗*	21.915 ± 9.226 *∗*^∧^	3.08 ± 2.539 ^∧^

*∗*There was a significant difference between the control group and the CCL4 treatment group since P < 0.05.

^∧^There was a significant difference between the CCL4 treatment group and CCL4/nAG treatment group since P < 0.05.

+There was a significant difference between the control group and CCL4/nAG treatment group since P < 0.05.

**Table 3:** The staging of liver fibrosis in the two experimental groups.

Degree of Fibrosis	Group II: CCL4 only	Group III: CCL4 and nAG
	(n=15 rats)	(n=15 rats)
No or mild fibrosis	2 (both rats had mild fibrosis)	9 (3 rats had no fibrosis and 6 rats had mild fibrosis)

Moderate to severe fibrosis	13 (4 rats had moderate fibrosis and 9 rats had severe fibrosis)	6 (4 rats had moderate fibrosis and 2 rats had severe fibrosis)

P= 0.008.

## Data Availability

Data are available at the College of Medicine Research Center, Deanship of Scientific Research, King Saud University, Riyadh, Saudi Arabia.
